# Early life ecology of the invasive lionfish (*Pterois* spp.) in the western Atlantic

**DOI:** 10.1371/journal.pone.0243138

**Published:** 2020-12-10

**Authors:** Jason Mostowy, Estrella Malca, Leif Rasmuson, Lourdes Vásquez-Yeomans, Trika Gerard, Eloy Sosa Cordero, Laura Carrillo, John T. Lamkin

**Affiliations:** 1 Marine Biology Department, Texas A&M University at Galveston, Galveston, Texas, United States of America; 2 Cooperative Institute for Marine and Atmospheric Studies, University of Miami, Miami, Florida, United States of America; 3 Southeast Fisheries Science Center, NOAA National Marine Fisheries Service, Miami, Florida, United States of America; 4 Marine Resources Program, Oregon Department of Fish and Wildlife, Newport, Oregon, United States of America; 5 Departamento de Sistemática y Ecología Acuática, El Colegio de la Frontera Sur, Chetumal, Quintana Roo, México; 6 University of Phoenix, South Florida Campus, Miramar, Florida, United States of America; 7 Departamento de Observación y Estudio de la Tierra, la Atmósfera y el Océano, El Colegio de la Frontera Sur, Chetumal, Quintana Roo, México; CSIR-National Institute of Oceanography, INDIA

## Abstract

The invasion of the western Atlantic by the Indo-Pacific lionfish (*Pterois volitans*/*miles*) is a serious threat to the ecological stability of the region. The early life history of the lionfish remains poorly understood despite the important role that larval supply plays reef fish population dynamics. In this study, we characterized patterns in the horizontal and vertical distributions of larval lionfish collected in the western Caribbean, US Caribbean, and the Gulf of Mexico from 19 ichthyoplankton surveys conducted from 2009–2016. Using generalized additive models (GAMs), we assessed the relative effects of spatiotemporal and environmental variation on the distribution of lionfish larvae. We also examined otoliths to determine larval ages and report the first larval growth rate estimates for this species. Lionfish larvae were present at 7.8% of all stations sampled and our model suggests that lionfish presence is related to sea surface temperature and the lunar cycle. Year and location also strongly affected the larval distribution, likely reflecting the ongoing expansion of the species during our sampling timeframe. Much of the variation in larval lionfish presence remained unexplained, and future studies should incorporate additional environmental factors to improve model predictions. This study improves our understanding of the lionfish life cycle and accentuates the need for further research into the early life history of this invasive species. The design and implementation of effective long-term lionfish control mechanisms will require an understanding of their entire life history.

## Introduction

Since the first documented sighting off the southeastern coast of Florida in 1985, the Indo-Pacific lionfish (*Pterois volitans*/*miles*) has spread throughout the tropical and subtropical western Atlantic, including the southeast coast of the United States, the Bahamas, the Gulf of Mexico, the Caribbean and the northeast coast of South America [1]. Subsequent sightings in the western Gulf of Mexico, the Windward Islands and the southwestern coast of Brazil indicate that lionfish are continuing to expand their range [2–4]. Lionfish have been documented in all near-shore marine habitats of the western Atlantic, including critical nursery habitats [5,6]. Coordinated removal efforts by spearfishing may provide a mechanism for invasion control on local scales, but the presence of lionfish on mesophotic reefs at depths exceeding recreational dive limits suggests that regional eradication of the species is not possible [7,8].

The success of the lionfish invasion has been largely attributed to the hardiness and adaptability of post-settlement lionfish, which appear to have few ecological constraints within the invaded range. They are generalist predators capable of consuming any prey within their gape limit and predate upon a diverse group of fish and invertebrate species [9–13]. Adult lionfish are tolerant of a wide range of biotic and abiotic habitat conditions, providing them with a large environmental niche in the western Atlantic [5, 14–16].

In contrast with the body of research focusing on the ecological dynamics of post-settlement lionfish, comparatively little is known about the early life ecology of these species. Lionfish exhibit a bipartite life cycle consisting of a benthic-associated, largely stationary adult phase and a pelagic planktonic larval phase capable of short- to long-range dispersal via ocean currents [17–19]. Lionfish are capable of reproducing throughout the year, with the largest individuals producing up to 42,000 eggs as frequently as once every 3 days [20, 21]. Fertilized egg masses float to the surface where larvae hatch, enter the pelagic environment, and disperse via surface ocean currents [22–24].

The successful colonization of the pelagic environment by larval lionfish can be inferred both from the rapid speed at which the invasion spread and from novel detections of lionfish larvae in pelagic habitats where they were previously absent [19, 24]. However, the specific dynamics of larval lionfish ecology have yet to be thoroughly examined. Marine larvae experience high mortality rates during the pelagic phase, and both mortality and somatic growth rates can be strongly influenced by ambient environmental conditions [25–28]. The physiological condition of a reef fish during the larval phase is positively correlated with their post-settlement survival rate, and individuals that encounter favorable environmental conditions as larvae are more likely to survive the subsequent post-settlement juvenile phase [29–31]. Thus, identifying the spatiotemporal and environmental parameters that mediate the distribution, survival and growth of larval lionfish will improve our ability to predict where and when lionfish will be prevalent in the zooplankton community.

In this study, we examined capture records from multiple ichthyoplankton survey efforts conducted throughout the tropical and sub-tropical western Atlantic with the goal of improving our understanding of lionfish larval ecology. We approached this task via three analytic objectives: 1) Characterize the horizontal and vertical distribution of lionfish larvae in the invaded range; 2) Use generalized additive models (GAMs) to quantify the relative influence of various environmental and spatiotemporal parameters on the probability of finding lionfish larvae in a given location; and 3) Examine larval otoliths to estimate larval ages and mean growth rates for a subset of lionfish.

## Methods

### Ethics statement

All surveys collected and handled ichthyoplankton in strict accordance with both international laws and those set by the United States Government 50 FR 20864 (May 20, 1985) [32]. Collections followed guidelines for the use of fish in research from the American Fisheries Society [33]. When conducting research in waters under the jurisdiction of nations other than the United States, research consent and permits for collection were initiated by the chief scientist and National Atmospheric and Oceanic Administration staff via the United States Department of State through normal diplomatic channels.

### Spatiotemporal distribution of larvae

#### Larval collections

**Fig 1:**
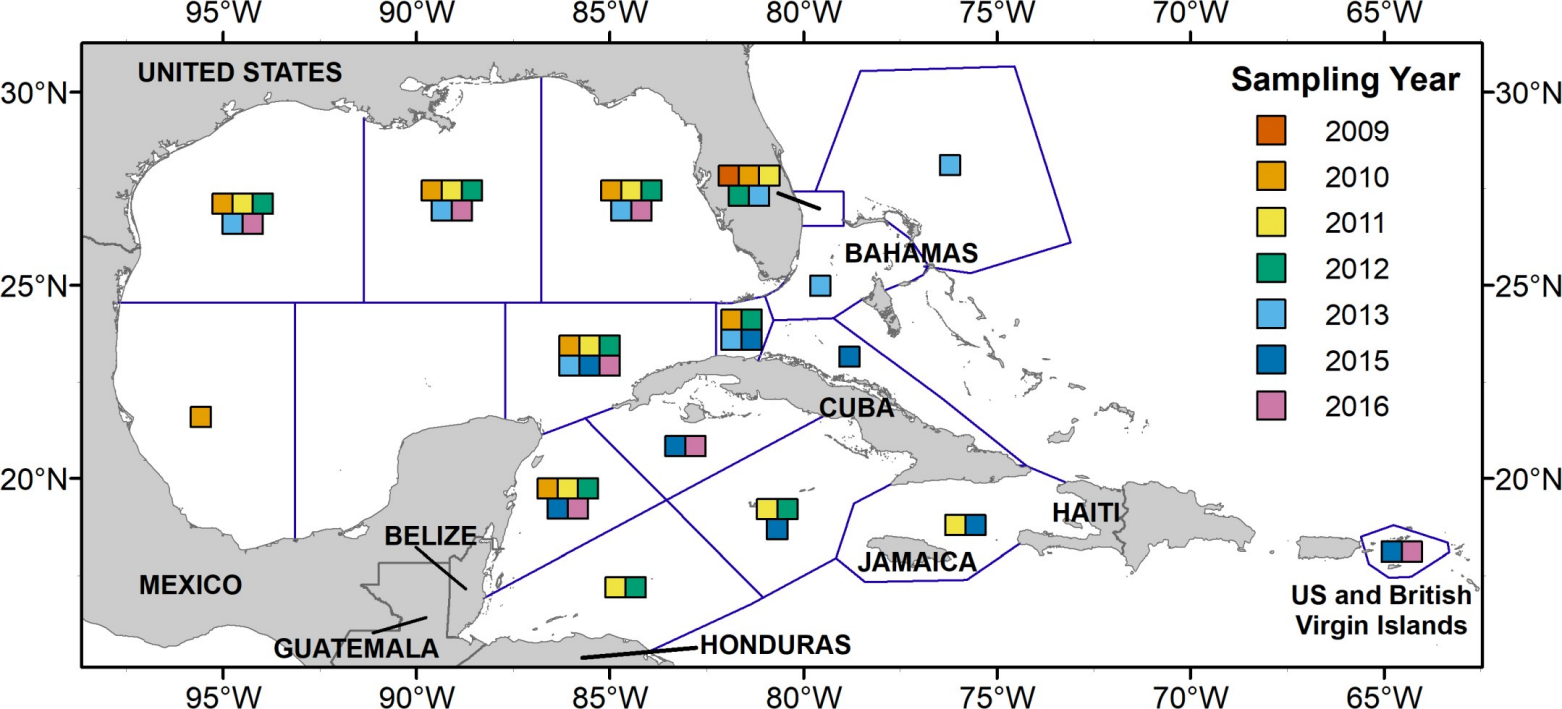
Location of sampling effort of the ichthyoplankton surveys compiled for this study. Blue lines demarcate general sampling regions, color swatches within a region denote the years that region was sampled from 2010–2016. Coastline data are sourced from [34].

Ichthyoplankton samples were collected during 19 surveys conducted between 2009 and 2016 aboard multiple research vessels (Table 1). The sampling area spanned approximately 16° to 30° N and 97°to 63° W, constituting a broad geographic range throughout the tropical and subtropical western Atlantic (Fig 1). Detecting larval lionfish was not the specific goal of any of the surveys; as such, sampling location, time of day, season and methodology varied among the datasets compiled for this study (Table 2). Spatiotemporal data, gear descriptions and environmental parameters are provided in S2 and S3 Tables. Samples were designated as daytime or nighttime based on the time of local sunrise and sunset for each survey. Ichthyoplankton were fixed in 95% ethanol that was replaced after 24 hours to ensure tissue preservation.

**Table 1:** Summary of the 19 ichthyoplankton collections and the corresponding regions surveyed in this study.

Year	Cruise Name*	Region Surveyed	n Stations Surveyed	n Stations with >1 Lionfish	% Stations with Lionfish Present	n Lionfish Collected	n Larval Fish Collected	Lionfish ‰
2009	WS0921	*Southeastern US*	9	1	11.1%	2	**794**	2.52
2010	GU1001	*Yucatan*, *NGoM*, *SWGoM*	199	4	2.0%	6	**63233**	0.09
	WS1009	*Southeastern US*	8	1	12.5%	3	**1076**	2.79
	NF1013	*EGoM*	73	2	2.7%	3	**41416**	0.07
	WS1016	*Southeastern US*	9	1	11.1%	2	**289**	6.92
	GU1004	*NGoM*, *EGoM*	145	4	2.8%	6	**57222**	0.10
2011	GU1101	*NGoM*, *W carib*.	231	42	18.2%	111	**117545**	0.94
	WS1109	*Southeastern US*	9	1	11.1%	1	**620**	1.61
	WS1114	*Southeastern US*	9	1	11.1%	1	**154**	6.49
2012	GU1201	*NGoM*, *W carib*.	208	33	15.9%	118	**135402**	0.87
	WS1206	*Southeastern US*	9	1	11.1%	1	**360**	2.78
	WS1210	*Southeastern US*	9	1	11.1%	3	**492**	6.10
	GU1204	*NGoM*	42	1	2.4%	1	**10966**	0.09
2013	ORII303	*NGoM*	104	3	2.9%	3	**55446**	0.05
	NF1304	*Southeastern US*, *Bahamas*	95	9	9.5%	15	**41416**	0.46
	PS1305	*NWGoM*, *EGoM*	162	4	2.5%	8	**78596**	0.10
2015	NF1502	*USVI*, *N*. *Cuba*, *W*. *Carib*.	274	29	10.6%	52	**154407**	0.34
2016	ORII317	*NGoM*	119	1	0.8%	1	**45611**	0.02
	NF1602	*USVI*, *N*. *Cuba*, *EGoM*	118	3	2.5%	4	**53110**	0.08
**Overall**			**1832**	**142**	**7.8%**	**341**	**858155**	**0.40**

*Cruise names are the first two letters of the ship utilized and the year sampled followed by the sequential survey number for each vessel. Lionfish ‰ is the number of lionfish larvae per 1000 fish collected in the sampling cruise. S1 Table provides additional details regarding each individual survey including dates sampled.

**Table 2:** Summary of the ichthyoplankton gear types deployed on the 12 surveys included in the model-fitting dataset.

Gear Name	Sampling Depth (m)	Volumetric	GU	NF	GU	GU	GU	GU	ORII	NF	PS	NF	ORII	NF	TOTAL
			1001	1013	1004	1101	1201	1204	303	1304	1305	1502	317	1602	
Neuston	0–0.5	No	199	65	145	214	149	42	103	14	157	0	114	8	**1210**
Bongo	0–200	Yes	117	33	138	49	43	42	104	38	162	0	119	69	**914**
MOCNESS	0–50	Yes	0	0	0	24	42	0	0	30	0	2	0	0	**98**
MOCNESS	0–80	Yes	0	0	0	0	0	0	0	0	0	0	0	10	**10**
MOCNESS	0–100	Yes	0	28	0	0	0	0	0	0	0	54	0	46	**128**
Surface 1	0–1	Yes	0	0	0	0	0	0	0	0	0	26	0	0	**26**
Surface 10	0–10	Yes	199	0	0	229	208	0	94	88	0	144	0	44	**1006**
Surface 25	0–25	Yes	0	0	0	0	0	0	0	0	0	0	0	60	**60**
Surface 50	0–50	Yes	0	0	0	0	0	0	0	0	0	149	0	0	**149**
**TOTAL**	-	-	**515**	**126**	**283**	**516**	**442**	**84**	**301**	**170**	**319**	**375**	**233**	**237**	**3601**

“Sampling Depth” indicates the depth range of the gear deployment; “Volumetric” column indicates whether the volume of water filtered was measured during the tow; Numbers within cruise columns indicate the number of deployments of each gear.

Ichthyoplankton were identified at the Larval Fish Lab in El Colegio de la Frontera Sur (Chetumal, Mexico), the Early Life History Lab at the National Marine Fisheries Service Southeast Fisheries Science Center (Miami, USA) and the Sea Fisheries Institute Plankton Sorting and Identification Center (Gdynia, Poland). The majority of the Gulf of Mexico (hereafter GoM) larvae were provided by the Southeast Area Monitoring Program for genus and species determination. Lionfish larvae were identified using morphological characteristics described in Imamura and Yabe [17] and Vásquez-Yeomans et al. [18]. Putative lionfish larvae that were smaller than 2 mm and which could not be genetically confirmed as *Pterois* spp. were omitted from counts and analyses, as species-specific morphological characteristics are not always reliable in identifying very early stage larvae. *Pterois* spp. larvae were classified into one of three developmental stages: preflexion, flexion or postflexion. Preflexion larvae were measured from the tip of the upper jaw to the end of the notochord. Flexion and postflexion larvae were measured from the tip of the upper jaw to the posterior midpoint of the caudal peduncle following Richards [35]. All body lengths (BL) were measured to nearest 0.05 mm (see S4 Table for detailed individual lionfish records). When volume filtered net data was available (volumetric tows), lionfish densities were calculated as the total number of lionfish divided by the total volume of water sampled (per 1000m^3^) during the net tow.

#### Vertical distribution analysis

We examined depth-stratified ichthyoplankton samples collected using a MOCNESS (Multiple Opening and Closing Net and Environmental Sensing System) to determine variations in larval lionfish density and BL with depth. MOCNESS deployments occurred during both day and night. All MOCNESS deployments were fitted with a 505μm mesh net and a built-in flowmeter to measure the volume of water filtered.

Three configurations (i.e. tow depth and vertical bin height) of the MOCNESS were used in the eight surveys that deployed a MOCNESS (Table 2). We selected the most prevalent configuration (MOCNESS 50) for statistical comparisons of larval lionfish density and BL between depth strata. The MOCNESS 50 sampled five depth strata: 0-10m, 10-20m, 20-30m, 30-40m and 40-50m. We omitted MOCNESS 50 tows where no lionfish larvae were collected at any depth, as the goal was to identify depth-specific trends where lionfish larvae were present. This left a vertical distribution analysis dataset of 29 MOCNESS 50 tows.

Because larval lionfish densities were not normally distributed, the Scheirer-Ray-Hare multifactor extension of the Kruskal-Wallis test [36] was used to evaluate significant variations in larval lionfish density by sampling depth bin and day vs. night. Post-hoc tests of significant results were conducted using pairwise-Wilcoxon tests with p-values adjusted via the Holm-Bonferroni method.

### Model of larval lionfish probability of presence

#### Oceanographic and environmental data sources

Potential explanatory variables were obtained from a combination of *in situ* environmental measurements, remote sensing and oceanographic models (Table 3). Temperature and salinity profiles were obtained using an SBE 9/11 plus CTD (conductivity/temperature/depth instrument) deployed at each station. Monthly-averaged surface chlorophyll-a concentrations were obtained from the Moderate Resolution Imaging Spectroradiometer-Aqua ocean color database [37]. Lunar phase data were obtained from the U.S. Naval Observatory Astronomical Applications Department [38]. Estimates of sea surface height, surface current direction and current magnitude at each sampling station were obtained from the Hybrid Coordinate Ocean Model (HYCOM, GBLa0.08 Expts. 90.8–91.2) [39]. Eddy kinetic energy was calculated from HYCOM estimates of zonal (u) and meridional (v) orthogonal current velocities using the equation:

$$EKE=\frac{1}{2}(u^{2}+v^{2})$$ (Eq 1)

**Table 3:** Name codes and descriptions of variables included in the GAM selection process and their data sources.

Variable name	Variable definition	Data Source
T5	Temperature at 5m, ^o^C	CTD
S5	Salinity at 5m, PSU	CTD
CHLA	Square root-transformed mean monthly surface chlorophyll concentration, mg/m^3^	[37]
LUN	Days since last new moon	[38]
EKE	Surface eddy kinetic energy, cm^2^/s^2^	[39]
SSH	Sea surface height, cm	[39]
DPTH	Bottom depth, m	[40]
DIST	Euclidean distance from shore, dec. deg.	[34]
TIME	Time of day	-
YEAR	Sampling year	-
MONTH	Sampling month	
LAT/LON	Latitude/Longitude	-

CTD indicates that variables were extracted from corresponding depth profiles sourced from the conductivity temperature depth sensor deployed at each station. Dashes (-) indicate variables sourced from shipboard logging systems.

Euclidean distance from the station to the closest shoreline was calculated using the GSHHG shoreline dataset [34]. Station seabed depth was extracted from the ETOPO1 Global Relief Model [40].

#### Model construction

GAMs (a non-parametric generalization of the generalized linear model) were selected for their flexibility in cases where the relationships between the explanatory variables and the response variables are nonlinear or not easily estimated *a priori* [41]. All models were constructed using the mgcv package in R [42].

The probability of larval lionfish presence at a given sampling station was modeled using a binomial distribution with a logit link function. A station was considered positive if at least one lionfish larva was detected in a volumetric tow at that station. None of the stations in 2009 could be included in the model-fitting dataset, as they lacked either volumetric tow information or relevant environmental data.

Stations in the GoM west of 90^o^W prior to 2011 were also omitted from the model-fitting dataset. Adult lionfish had not yet been recorded in that area prior to 2011 [1], nor were their larvae detected in the area in this study prior to 2011; this raised concerns that including these stations in the GAM fitting process would bias the resulting models with false zeros, especially given the inherent risk of overfitting when using GAMs [43]. The resulting models should not be considered to apply to areas west of 90^o^W, prior to 2011.

Strong correlations between potential explanatory variables were managed using a modification of the methods of Rooker et al. [44]. A Spearman’s ρ correlation matrix comparing all potential explanatory variables was constructed. In cases where the absolute rank correlation between two or more variables was greater than ρ = 0.6, single variable GAMs were constructed for each variable in question and the Akaike Information Criterion (AIC) [45] of each model was calculated. The explanatory variable that generated the model with the lowest AIC was included in the model selection process.

Once the set of non-correlated explanatory variables was identified for the dataset, a model selection process was conducted using an exhaustive search method. A set of all possible models consisting of every combination of explanatory variables was constructed, and the resulting models were ranked by increasing AIC. Smooth functions for most explanatory variables were restricted to 4 degrees of freedom to avoid overfitting, while smooth functions for cyclic variables (e.g. sampling time and days since the new moon) were restricted to 6 degrees of freedom. Sampling effort at each station was standardized by including the log-transformed volume of water filtered at the station as an offset term in the model. A bivariate spline of latitude and longitude was included in each model to account for broad scale spatial variation in larval distributions [46]; this bivariate spline was not restricted to a particular number of degrees of freedom.

Candidate models were selected from a list of all possible models based on the following criteria: 1) Parsimony, with less complex models being preferred over those with more terms; 2) The magnitude of the decrease in likelihood (increase in AIC) of the candidate model relative to the most likely model (ΔAIC), where a ΔAIC difference > 2 was considered to be a significant loss in model support [47]; and 3) Ecological explicability of the included variables, with models having a simple ecological explanation being preferred over models with more complex interpretations.

#### Model evaluation

The final classification model was evaluated by determining a bootstrapped mean area under the receiver operating characteristic curve (AUC) using the ROCR package in R [48]. In each iteration, the full dataset was randomly split into a training set consisting of 70% of the original data and a testing set with the remaining 30% of the original data, and the variable smoothing functions from the final model were re-fitted to the training dataset. The refit model was used to predict the response probability for each station in the testing dataset. A receiver operating characteristic (ROC) curve was generated from the set of response predictions and its corresponding AUC was calculated. The sensitivity and specificity of the model over the testing set predictions were also calculated. This process was repeated 10,000 times to generate mean AUC, sensitivity and specificity of the mode. The results were evaluated according to the AUC cutoff criterion suggested by Hosmer et al. [49] where an AUC of 0.7–0.8 = Acceptable, 0.8–0.9 = Good and > 0.9 = Outstanding.

### Genetics and ageing

A subset (n = 88) of larvae collected in the GU1101 survey (March—May 2011) from the western Caribbean was selected for genetic analysis to confirm visual identifications. Approximately 1mm^3^ of larval muscle tissue was removed with fine sterilized tweezers. The mitochondrial cytochrome c oxidase 1 gene (MTCO1) amplification and sequencing methodology is the same used in Vasquez-Yeomans et al. [18]. Reference vouchers were deposited in El Colegio de la Frontera Sur, Chetumal Unit. DNA extraction and sequence analyses were carried out in Duke University’s Marine Laboratory in Beaufort, North Carolina (T. Schultz, pers. com.).

In addition, tissues from two larval lionfish vouchers (ECO-CH-LP 5283 and ECO-CH-LP 16339) were also extracted and amplified with C-Fish cocktail [50]. Voucher sequences were edited using CodonCode v.3.0.1 (CodonCode Corporation, Dedham, MA) and uploaded to the Barcode of Life Data System (boldsystems.org) in the dataset DS-LFLAR. All data were analyzed with BOLD, and all sequences were examined for the presence of stop codons [51].

A second subset of larvae (n = 60) from the GU1101 survey were selected for ageing. Larvae selected for this subset spanned a representative size range, and the smallest specimens (< 2 mm) were genetically confirmed as *Pterois volitans* prior to ageing. Sagittal otolith removal and ageing methods follow Malca et al. [52]. Digital images of each sagitta were captured at 400x to 1000x using transmitted light and a compound microscope (Axio A.1, Zeiss) equipped with a digital camera (Micropublisher 3.3 RTV, Qimaging). One sagitta was randomly selected and two experienced readers independently enumerated increments along the longest axis (otolith radius, OR). Image analysis software (ImagePro Plus 7) was used to enumerate and measure individual increment widths using the Otolith Macro ^TM^, (Media Cybernetics, Inc.) along the OR. The coefficient of variation (CV) measured the precision between readers [53]. Mean growth rates (mm/day) were calculated using BL divided by the number of increments. Increments were assumed to occur daily and were not corrected. Hatching dates were estimated by subtracting the estimated daily increments from the collection date. A simple exponential growth curve was also fit to the final age-BL data:

$${BL}_{it}={BL}_{0}\times e^{KX_{t}+\varepsilon_{i}}$$ (Eq 2)

Where BL_*it*_ = body length of fish *i* at day t, L_*0*_ = body length at age 0, K = instantaneous growth coefficient, X_t_ = age in days, and *ε*_*i*_ is an error term on the measurement of fish *i*.

## Results

**Fig 2:**
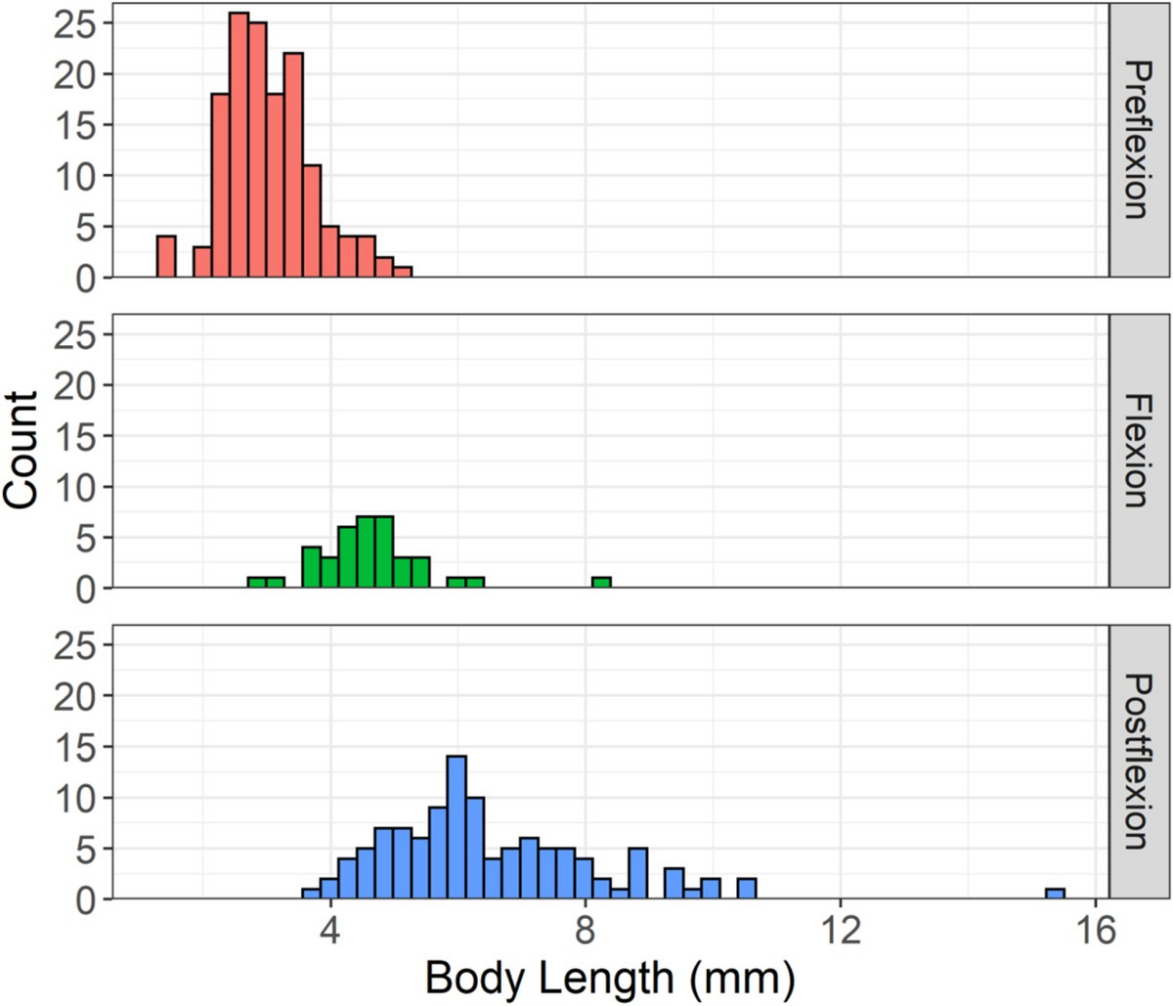
Histogram of lionfish (n = 341) body length (mm) shown for each of the larval developmental stages (preflexion [n = 145], flexion [n = 112] and postflexion [n = 46]) from all gears.

In total, 341 lionfish larvae were collected during the 19 surveys analyzed in this study (Table 4). Of these, 43% (145) were preflexion, 11% (38) were flexion and 33% (112) were postflexion. 13% (46) of the larvae were too damaged to be assigned a developmental stage. Mean BL and their 95% confidence intervals were 3.03 ± 0.11 mm, 4.64 ± 0.30 mm and 6.54 ± 0.33 mm for preflexion, flexion and postflexion larvae, respectively (Fig 2).

**Table 4:** Summary of lionfish frequency of occurrence and larval density by geographic region.

Region	Years Surveyed	n Stations	n Lionfish Larvae	% Freq. of Lionfish	Mean Lionfish Density (ind./1000m^3^)
Western Caribbean	2010, 2011, 2012, 2015, 2016	384	230	21.1%	0.38
Gulf of Mexico	2010, 2011, 2012, 2013, 2015, 2016	1097	44	2.4%	0.05
Southeastern US, Bahamas	2009, 2010, 2011, 2012, 2013, 2015	208	51	12.0%	0.61
US and British Virgin Islands	2015, 2016	143	16	7.0%	0.11
**Overall**	-	**1832**	**341**	**7.8%**	**0.36**

Density was calculated as the number of lionfish divided by the total volume of water sampled in the region (per 1000m^3^).

### Spatiotemporal distribution of lionfish larvae

#### Horizontal distribution

**Fig 3:**
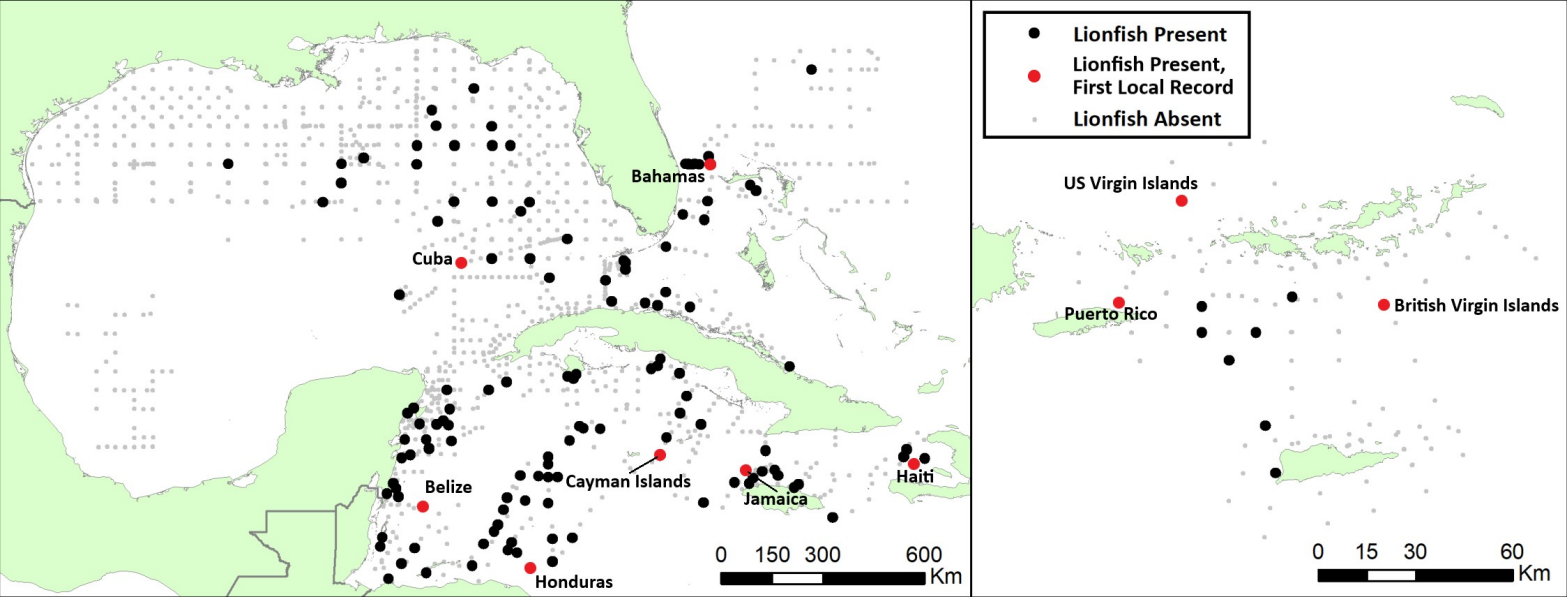
Larval lionfish presence/absence at all compiled stations. (Black bullets) denotes larval lionfish presence; (Red bullets) denotes first larval lionfish record for labeled countries/territories; **a)** Stations from the western Caribbean, Gulf of Mexico, Southeastern United States and Bahamas; **b)** Stations surveyed in the US Caribbean and British Virgin Islands. Coastline data in this map are sourced from [34].

**Fig 4:**
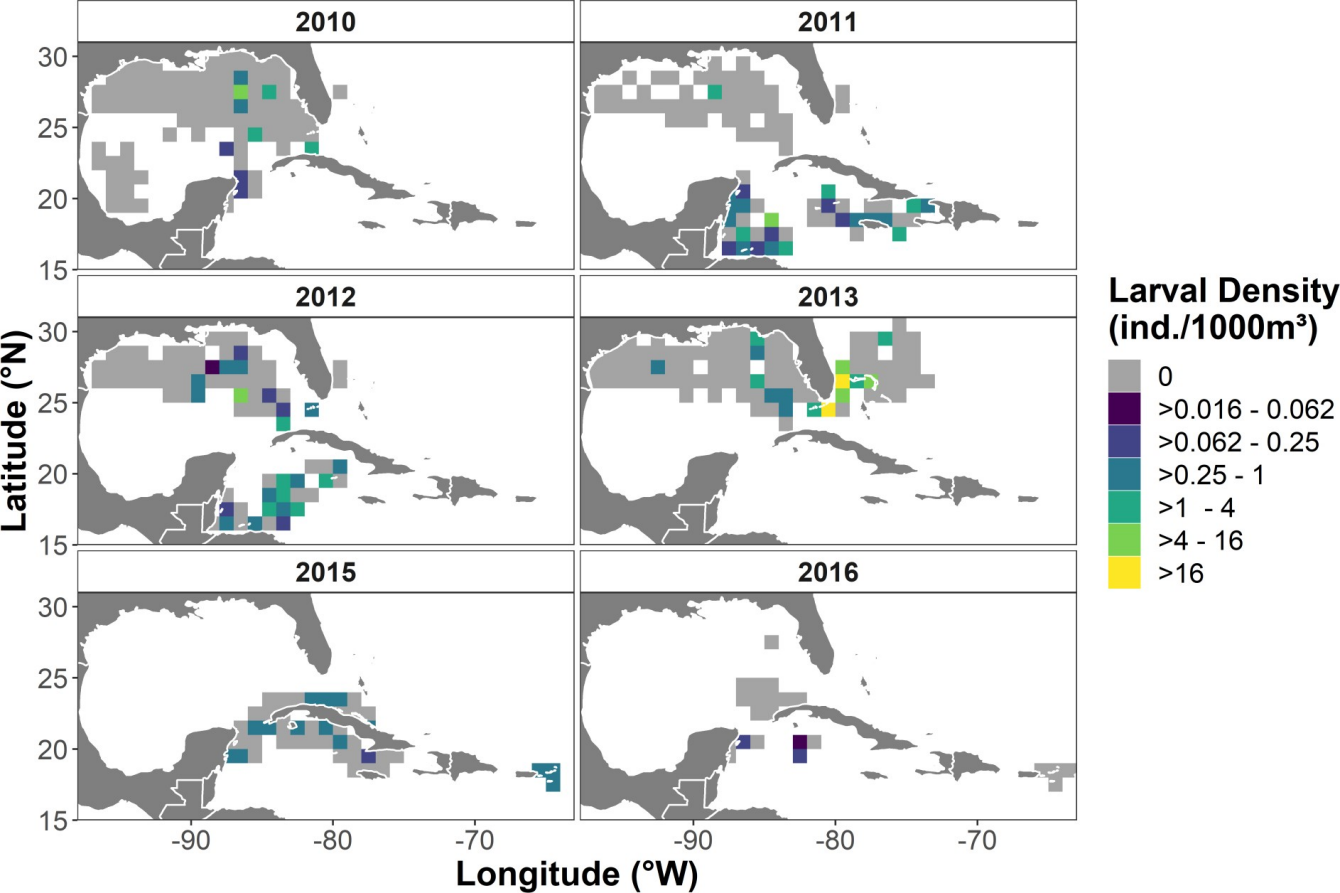
Larval lionfish density (ind.·1000m^-3^) visualized over a 0.5^o^ x 0.5° cell grid. **a**) 2010, **b**) 2011, **c**) 2012, **d**) 2013, **e**) 2015, and **f**) 2016; Cell value represents the total number of lionfish detected at all stations within the cell, divided by 1/1000^th^ of the total volume of water sampled in the cell. Blank (white) regions were not sampled in a given year. Note the logarithmic scale. Coastline data in this map are sourced from [34].

Lionfish larvae were present at 142 of the 1832 stations sampled (7.8%), and the distribution of lionfish presence varied by both year and geographic region (Fig 3). Larvae were detected at 21.1% of western Caribbean stations and 7.0% of the stations in eastern Caribbean in the US and British Virgin Islands region. In the GoM, lionfish were collected at 2.4% of the stations sampled, almost exclusively in the eastern portion of the basin with the exception of a single station in 2013. Lionfish were detected at 12.0% of stations near the southeastern US and Bahamas. Lionfish larvae were most frequently detected (17.7% of stations) during 2011, when surveys targeted the western Caribbean. They were least frequent (2.8% of stations) in 2010, when sampling was most intensive in the GoM. Larval densities also showed a wide range of spatiotemporal variation (Fig 4). The mean density of lionfish larvae was 0.36 ± 0.15 ind.·1000m^-3^ over all volumetric samples collected (Table 4). Peak densities were observed off the Florida coast and the upper Bahamian archipelago in 2013, where mean larval densities were 9.19 ± 7.11 ind.·1000m^-3^. The maximum single-station density occurred at a station in the Straits of Florida in 2013, where the larval lionfish density was 57.14 ind.·1000m^-3^

#### Vertical distribution

**Fig 5:**
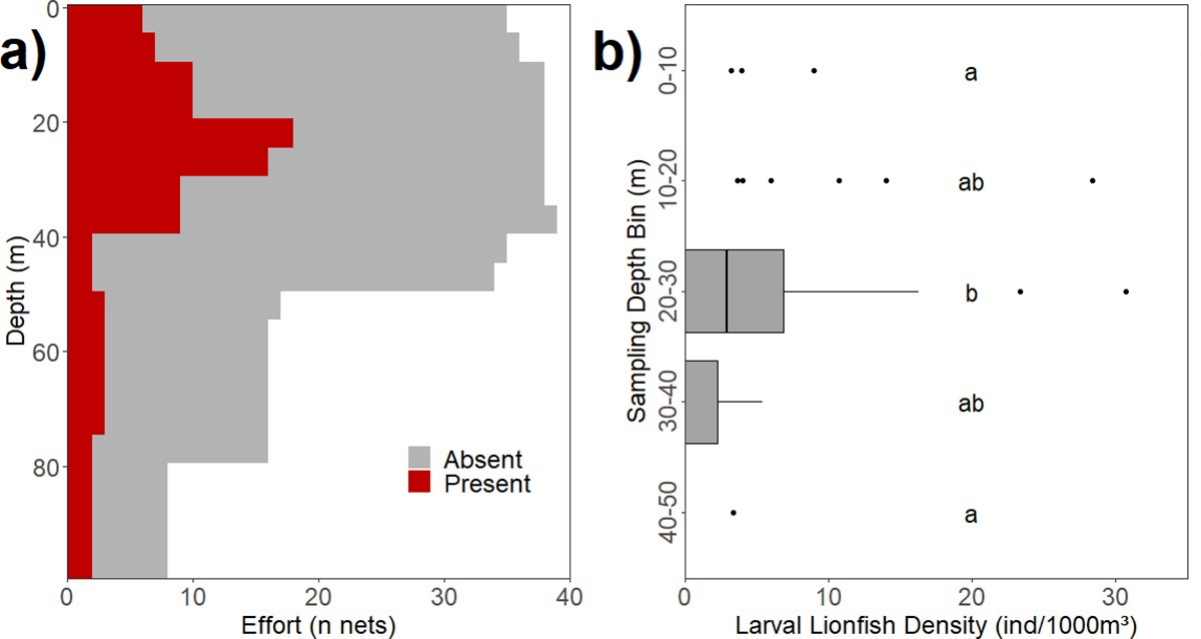
Vertical distribution of lionfish larvae from MOCNESS tows. **a)** Vertical distribution as the number of lionfish-positive nets vs. sampling depth (m); **b)** Larval lionfish density (ind. 1000m^-3^) in each of the depth bins from the MOCNESS 50 tows. Letter groups **(a, b)** denote bins where density did not significantly differ (α = 0.1); Letter groups **(a, b)** denote bins where length did not significantly differ (α = 0.1).

Lionfish larvae were distributed throughout the water column from the surface to the deepest depth bin sampled (75-100m) (Fig 5A). Larvae were more common in the upper 50m of the water column; at stations where larvae were present in a MOCNESS tow, larvae were found in 24.4% of nets towed in the top 50m of the water column compared with 12.5% of nets below 50m.

Within the 0-50m MOCNESS tows, larvae were most frequently captured in the 20-30m depth bin (Fig 5A). The Scheirer-Ray-Hare test suggested that effect of sampling depth on larval lionfish density was significant (p = 0.0006), while day/night and the interaction term had no significant effects. Post-hoc pairwise-Wilcoxon tests indicated that lionfish densities were significantly higher in the 20-30m bin than in the 0-10m (p = 0.02) and 40-50m (p = 0.003) depth bins (Fig 5B).

### Model of larval lionfish probability of presence

The final modeling dataset included 1274 sampling stations. Of the 1832 ichthyoplankton station records collected in this study, 408 stations (22.2%) lacked data either for an environmental variable or for the volume of water sampled at the station. These stations were omitted from the model-fitting dataset. Lionfish larvae were present at 114 (8.9%) of the stations in the final model-fitting dataset.

The model selected to explain the probability of larval lionfish presence retained 4 variables in addition to the bivariate spline of latitude and longitude: sampling year (YEAR), days since the previous new moon (LUN), time of day (TIME), and temperature at 5m (T5, Table 5). The model had the form:

$$logit\left(p_{i}\right)=te\left(LON_{i},LAT_{i}\right)+offset_{i}+s\left(LUN_{i}\right)+s\left(TIME_{i}\right)+s\left(T5_{i}\right)+YEAR_{i}$$ (Eq 3)

**Table 5:** Summary of the final GAM of larval lionfish presence.

**Factor Level**	**Estimate**	**Std. Error**	**p-value**
Year 2010	-9.1805	0.3958	< 0.0001*
Year 2011	-9.4760	0.3345	< 0.0001*
Year 2012	-9.2416	0.3166	< 0.0001*
Year 2013	-8.8877	0.4649	< 0.0001*
Year 2015	-10.1628	0.3633	< 0.0001*
Year 2016	-13.1248	0.7692	< 0.0001*
**Smooth function**	**ΔDE (%)**	**edf**	**p-value**
Days since new moon	0.4%	2.516	0.001 *
Sampling time	0.9%	1.440	0.043 *
T5	0.9%	2.897	0.003 *
Spatial spline	8.0%	9.003	0.001 *
Year	4.3%	-	-

Top panel indicates the effect of each level of the year factor (2010–2013, 2015, 2016) on logit (probability of presence) while the lower panel shows the estimated significance levels of the smooth functions. ΔDE is the loss in percent deviance explained caused by dropping the variable; “edf” is the estimated degrees of freedom for smooth terms

(*) denotes statistical significance (α = 0.05).

Where *p*_*i*_ is the probability of larval lionfish presence at station *i* and offset_i_ is the offset term for station *i*, a weighting term determined by the natural-logged volume of water sampled at station *i*.

**Fig 6:**
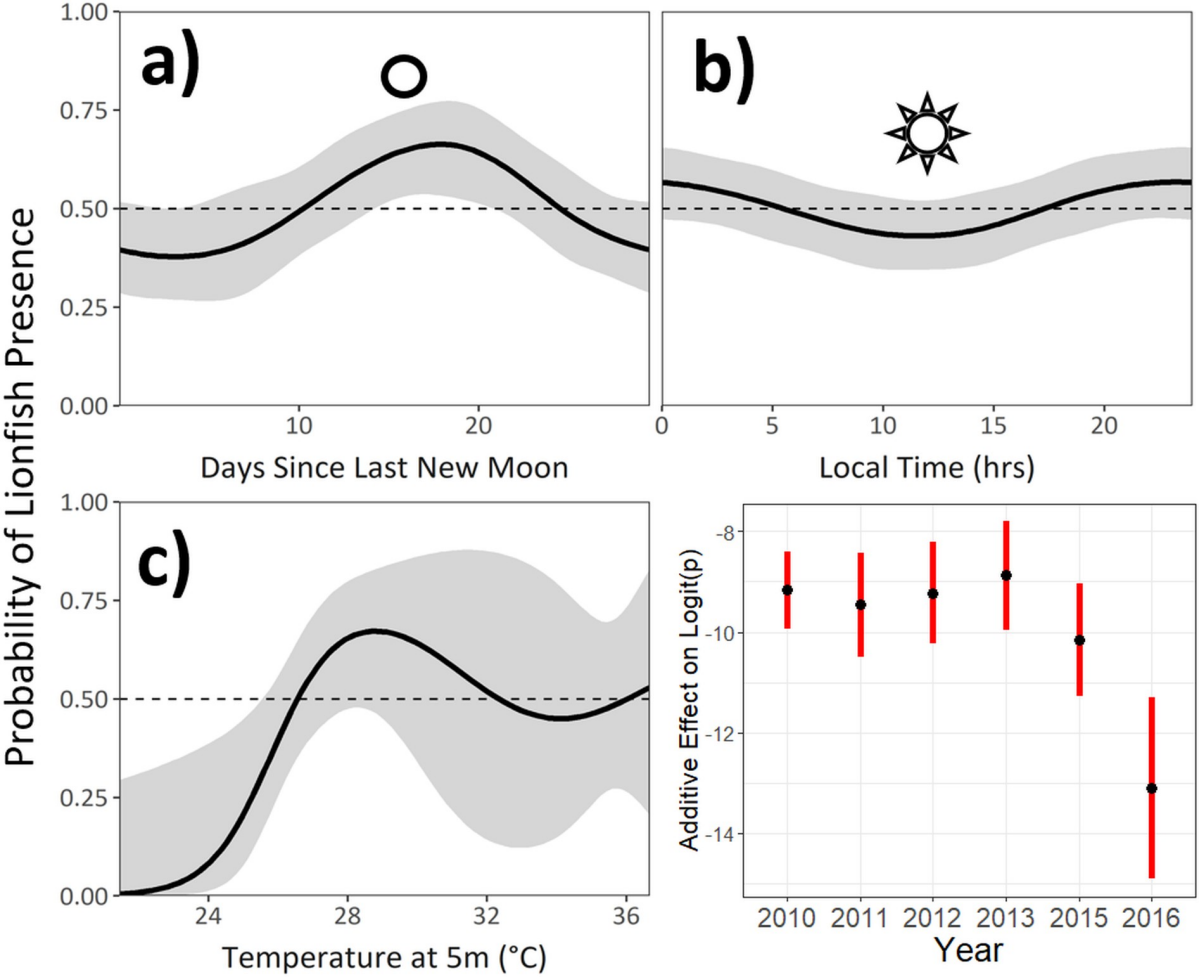
Smooth functions of the final GAM of larval lionfish presence. **a)** Lunar phase, open circle denotes the full moon; **b)** Sampling hour, sun symbol denotes local noon; **c)** Temperature at 5m; **d)** Effect of the year factor.

**Fig 7:**
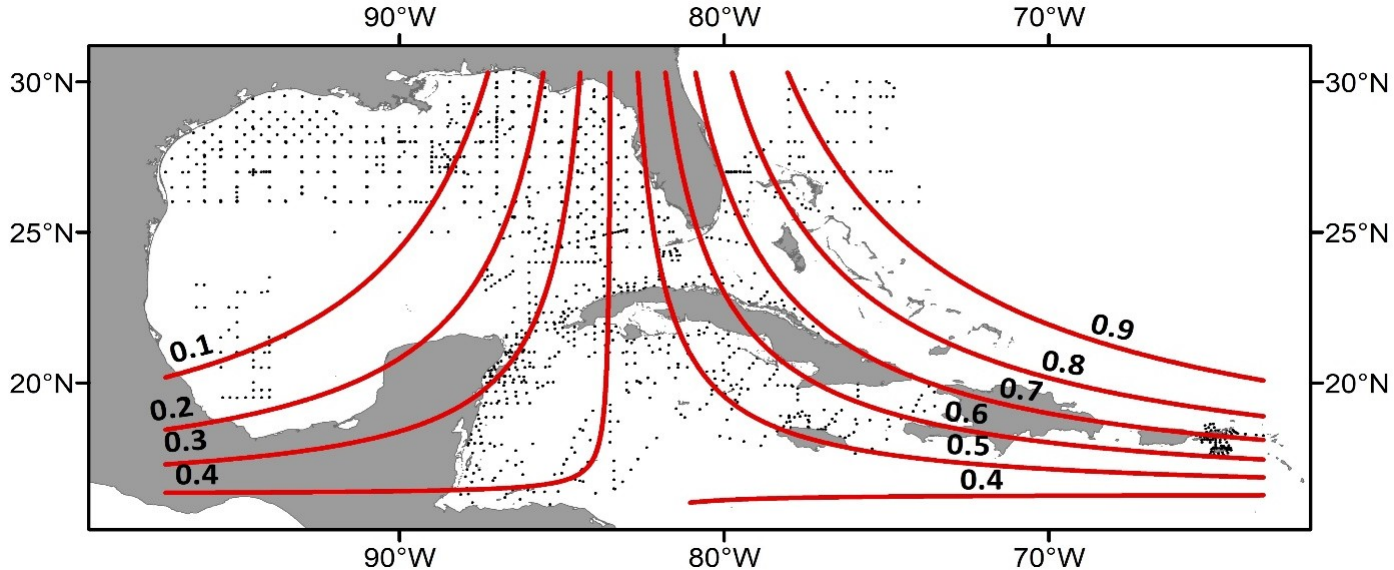
Simulated effect of the bivariate longitude/latitude spline on larval lionfish probability of presence. All ichthyoplankton stations sampled in this study (2009, 2010, 2011, 2012, 2013, 2015 and 2016) are plotted. Red contours show lines of constant detection probability generated by inputting simulated data into the model and evaluating the result across a grid of latitude and longitude values. Coastline data are sourced from [34].

The final model explained 18.6% of the residual deviance and had a bootstrapped mean AUC of 0.78 ± 0.03 (acceptable). The probability of lionfish presence showed a roughly parabolic relationship with T5, with a maximum probability of presence at approximately 29°C (Fig 6). The relationship between lionfish presence and sampling time was also parabolic, with the highest probability of presence at midnight and a minimum near local noon. Lionfish presence showed a peak approximately 4 days after the full moon, with the lowest probability of presence near the new moon. The effect of year was significant for all sampling years. In general, the spatial spline showed increasing lionfish probability from north to south and west to east (Fig 7).

### Genetics and ageing

Of the 88 larvae sent for mtDNA identification, 69 were confirmed as *P*. *volitans* (T. Schultz, pers. com.). Within this subset, 12 preflexion larvae (~1–3 mm BL) provide the smallest specimens confirmed with genetics. Although mtDNA barcoding was unsuccessful for 19 larvae, these specimens were morphologically similar and it is likely that insufficient material was isolated for adequate analysis. The mtDNA sequence of the two voucher specimens (ECO-CH-LP 5283 and ECO-CH-LP 16339) were submitted to GenBank (Accession No. MT048384 and JN312282 respectively) and uploaded to BOLD, where they are available in the dataset Lionfish Larvae (DS-LFLAR; http://www.boldsystems.org/index.php/Public_SearchTerms?query=DS-LFLAR).

**Fig 8:**
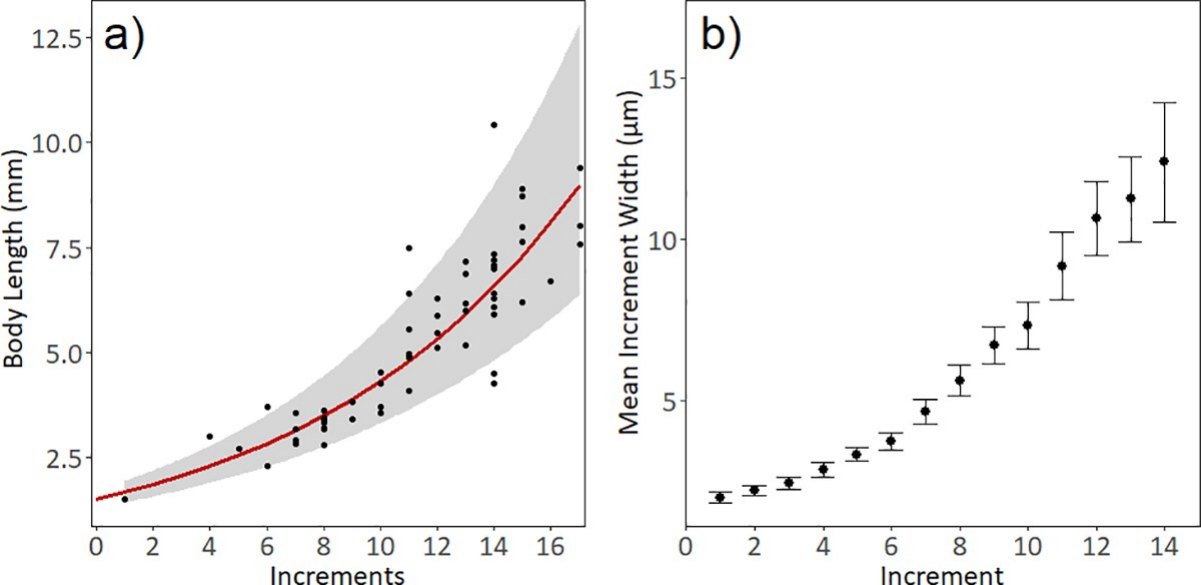
Larval lionfish ageing results from otolith analysis. **a)** Larval size-at-age from subset of lionfish collected in March, April and May 2011, red line shows the regression of ln(Body Length) on daily Increments (BL = 1.51e^0.105*n^, R^2^ = 0.83), with a 95% CI; **b)** Mean otolith increment widths with 95% CI.

Lionfish were 1 to 17 days old with a mean age of 11 days (Fig 8A). Aged lionfish ranged from 1.5 mm to 10.42 mm and included larvae in all three developmental stages. The growth equation for lionfish larvae was an exponential curve BL_mm_ = 1.484 e ^0.105x^, where BL_mm_ = body length (mm), and x = age (days). The majority (71%) of the 60 larvae aged, including the two smallest larvae (< 2 mm notochord length), were genetically confirmed as *P*. *volitans* (T. Schultz, pers. com.). Acceptable agreement between both readers (CV < 15%) was observed for 56 of the 60 otoliths aged while 4 larvae were discarded with high CVs. No significant differences were found between ANOVA comparisons of age estimates between reader 1 and reader 2 (F_1,116_ = 0.2994, p = 0.915), therefore one randomly chosen read amongst the four increment counts was selected to be the representative age for each larva. Increment widths ranged from 2 to 10 μm and increased rapidly with larval age (Fig 8B). The back-calculated hatching dates for aged larvae start in 17 March to 19 April 2011. The two larvae collected in the GoM in 24 May 2011 were 13 and 14 days old and were estimated to hatch on 10 and 11 May 2011, respectively. The OR ranged from 9.2 to 154.1 μm and showed a linear relationship with body length (BL_mm_ = 0.0589 x OR_μm_ + 1.3785, R^2^ = 0.9533).

## Discussion

The 341 larvae reported in this study represent the most comprehensive larval lionfish collection to date in the invaded range and includes multiple first larval records for the western Atlantic region (Fig 3). The first larval lionfish collections are reported in the waters of the Bahamas, Belize, British Virgin Islands, Cuba, Haiti, Honduras, Jamaica, the Cayman Islands and in the US Caribbean (Puerto Rico, St. Thomas, St. John and St. Croix). Although sampling effort was concentrated during March to June, larval collections are also reported for the first time in August through November.

### Spatial distribution of lionfish larvae

#### Horizontal distribution of lionfish larvae

The distribution of lionfish larvae was inconsistent in space and time, but the aggregated larval densities roughly correspond to the regional sequence of the overall invasion. The invasion has progressed in three main stages: an initial spread along the US southeastern Atlantic coast and into the Bahamas from 1985–2006, a radiation through the Caribbean from 2006 onwards and a current-driven movement into the GoM beginning in 2010 [1, 54]. We found the highest larval densities off the southeastern Florida coast and the Bahamas, while intermediate densities were generally observed in the Caribbean, and larvae were comparatively scarce in the GoM (Fig 4). A similar geographic pattern can be noted in the 2-D spatial spline included in the final model, where larval presence was increasingly less likely moving clockwise from Florida to the GoM (Fig 7).

The scarcity of lionfish larvae in the northern GoM (nGoM) during sampling in the spring and summer of 2013 is particularly interesting, as lionfish were well established throughout the nGoM by 2013 [1, 55], often in high densities [56]. Larval lionfish were almost entirely restricted to the eastern GoM (Fig 3), which was the first area of the basin to be invaded [57]. A possible explanation would be a lag between the establishment of a lionfish population in an area and the ability of that population to reproduce at detectable levels. At the time of sampling in 2013, the lionfish population in the nGoM had only been established for approximately 3 years and was still rapidly growing in density [1, 56]. As the lionfish in the GoM reached maturity at age 1 [58], the nGoM population may have lacked enough reproductively mature individuals for their larvae to be detected in 2013. In addition, the lack of larvae in the nGoM may be influenced by the comparatively high densities of lionfish on nGoM artificial habitats compared with other invaded habitats [56]. High densities have been linked to decreased body condition in adult lionfish [59], which in turn can decrease the number and quality of offspring produced by reef fish [60]. However, Muhling et al. [61] found that the overall abundance of scorpionfish (Scorpaenidae) larvae in the nGoM was more strongly associated with eastern longitudes than with any other factor examined; as such, the geographically skewed distribution of lionfish larvae we observed in the GoM may not be unique to *Pterois* spp. but rather may be a general characteristic of GoM scorpaenid larvae. Finally, fish larvae transport pathways are neither linear nor direct from hatching to suitable reef habitats, because there is variability in the main circulation such as extension/retraction of the Loop Current into the Gulf, meanders, shears, frontal eddies and detachment of anticyclonic eddies that travel westward [62], that may result in a non-homogeneous and scarce larvae distribution in the oceanic waters of the GOM. In general terms, the chronology of the lionfish invasion [1] roughly reflects the circulation pattern that is part of the North Atlantic Ocean western boundary current system, moreover, the transport of larvae into the nGOM via the Loop Current as hypothesized by Kitchens et al. [19] suggests that larvae sources may be located upstream. This could explain the increased detection of lionfish larvae in eastern longitudes.

Although larval lionfish densities were variable in this study, our collections indicate that they now compose a considerable portion of the ichthyoplankton community in certain parts of the invaded range. Sponaugle et al. [24] reported lionfish densities of 0.4–0.7 ind./1000m^3^ at stations in the Florida Keys and Straits of Florida, concluding that lionfish composed 13–26% of the scorpaenid community in the ichythoplankton. Mean densities in this study for a similar area were a comparable 0.8 ind./1000m^3^. Although collections from the Straits of Florida in this study predated those of Sponaugle et al. [24] by a year, there was seasonal overlap in collection times. Comparisons in other invaded areas also suggest lionfish larval densities may be significant relative to native species. Muhling et al. [63] found that in 2006 and 2007, the Mesoamerican Barrier Reef System larval fish communities showed mean combined scorpaenid densities of 0.6 ind./1000m^3^, prior to the main lionfish invasion of the region (reanalyzed unpublished data). In this study we found lionfish densities of 0.29 ± 0.08 ind./1000m^3^ in the same area. Barring a large change in the larval density of other scorpaenid species in the intervening years, these results suggest lionfish likely constitute one of the largest single-species contributors to the scorpaenid larval community in the area. While densities in the native range have not been reported, larvae from all *Pterois* species combined account for up to 12.5% of the total scorpaenid larvae from studies conducted in the native range of the *Pterois* genus [64]. Relative percentages in the invaded range appear to have a lower bound near this native maximum. The degree to which other fish larvae compete with larval lionfish for food resources is unknown, as specific diets of lionfish larvae have yet to be examined. Larval fish at lower latitudes tend to have more specialized diets compared with temperate larvae [65], and native species with spatiotemporal and dietary niches that overlap that of larval lionfish would face particularly strong competition.

#### Vertical distribution of lionfish larvae

Our findings suggest that lionfish larvae exhibit some capacity for controlling their vertical position in the water column. Overall larval densities were significantly higher in the 20-30m MOCNESS depth bin than all other depth bins (Fig 5A). This finding agrees with those of Sponaugle et al. [24], who found lionfish larvae were concentrated at similar depths. Scorpaenid larvae have characteristic robust fin elements during preflexion stage, and *Pterois* spp. is no exception. Robust fins facilitate swimming behavior that can allow fish larvae to remain in favorable pelagic habitat or orient themselves towards potential settlement areas [66, 67]. In particular, vertical motion has important implications for reef fish population connectivity, as reef fish larvae have been shown to adjust their mean depth to promote retention near high-quality natal habitats and restrict their dispersal distance [68, 69]. The two largest lionfish in our collections measured 15.33 mm and 15 mm respectively, however only a few larvae were larger than 10 mm despite extensive net tows. Net avoidance by these larger (>10mm) specimens is likely in addition to their ability to retain some preferred depth strata.

### Model of larval lionfish probability of presence

Spatiotemporal variation, namely sampling year and longitude-latitude, accounted for the majority of the residual deviance explained in the final model of larval lionfish presence (Table 5). Previous attempts to model the spread of the lionfish invasion found that the dominant current regime most effectively explained the spatial pattern by which the invasion progressed [54]. Our model results support this, as incorporating environmental variation only slightly improved the explanatory power of the model. However, the retained environmental parameters were capable of providing additional insights even after the spatiotemporal variability had been accounted for. The probability of larval lionfish presence showed a threshold-like relationship with temperature at 5m, with a maximum at ~29°C. Warmer water temperatures are associated with increased metabolic rates, decreased pelagic larval durations and faster somatic development in reef fish larvae. This may reflect a developmental strategy in the western Atlantic that favors rapid larval growth in the first weeks of life [70, 71]. Larval lionfish in this study grew faster when compared with some native reef fish species, but the precise reasons for the observed increased probability of larval presence in warmer waters merits additional investigation.

Lunar phase also had a significant effect on the probability of detecting a lionfish larva. Many reef fish use lunar cues to synchronize spawning events [72, 73]. The lunar phase spline shows a maximum at 18 days, shortly after the full moon. This peak in probability could be explained by synchronized spawning by lionfish at the first quarter moon or in the lead-up to the full moon. Other mid- to large-bodied reef fish species show a similar pattern of lunar synchronicity in spawning, although in many cases it also involves an aggregatory behavior [74]. Histological evidence suggests that lionfish are asynchronous batch spawners capable of spawning year-round [20], and while seasonal effects on lionfish fecundity have been reported [21, 58], the influence of lunar periodicity on spawning potential has yet to be addressed. The observed peak in probability suggests some correlation of lunar phase with larval presence, although further research is needed to determine if this pattern is caused by the spawning behavior of the adults or by a behavioral response of the larvae to enhance their survival during their pelagic phase. Positive phototropism is anecdotally reported in *Pterois* larvae and more robustly reported in the larvae of some related scorpaenids [75], and could constitute a mechanism for a larval behavioral response to varying lunar illumination, if such a response exists.

While the influence of sampling time on larval presence was relatively weak (ΔDE = 0.9%, Table 5), its effect showed a clear periodic pattern with a maximum at approximately midnight and a minimum at local noon (Fig 6). Two behavioral responses may account for the observed diel periodicity in larval presence. First, larger and more developed larval fish are capable of detecting and avoiding sampling nets, particularly during the daytime when ichthyoplankton gear is more visible [76]. Second, many planktonic species undergo diel vertical migrations, which have been hypothesized to promote a favorable balance between prey density and encounters with predators [77, 78]. If lionfish larvae are adjusting their depth diurnally, then the observed decrease in larval presence during daylight hours may reflect a deeper daytime distribution in larvae.

### Larval lionfish age and growth

Growth rates for the lionfish larvae aged in this study were more similar to pelagic taxa such as istiophorids and swordfish than to other similar-sized reef fishes. The mean instantaneous growth coefficient K was 0.105, comparable to that of larval blue marlin (*Makaira nigricans*, K = 0.085–0.128) from the Bahamas and Straits of Florida [79] and to larval sailfish (*Istiophorus platypterus*, K = 0.144) from the nGoM [80]. In contrast, the larval growth coefficient of western Atlantic snappers such as schoolmaster (*Lutjanus apodus*, K = 0.047) and mutton snapper (*Lutjanus analis*, K = 0.044) are considerably lower [81].

The first reported lionfish larva in the invaded region was collected in April 2010 (ECO-CH-LP 5283) [18]. This larva measured 8mm BL and was estimated to be 15–16 days old, which aligns with our observed growth curve (Fig 8A). Two GoM larvae collected in 2011 were also aged, and their growth trajectory was similar to the western Caribbean larvae. However, larvae collected in the nGoM in 2011 by Kitchens et al. [19] appear to be growing faster than those in this study. Temperature may play a role in this discrepancy. Larvae in the Kitchens et al. [19] study were collected in warmer waters (approx. 29 ˚C) in June and July, while most of the larvae aged in this study were collected in the western Caribbean in spring 2011 where mean 5m water temperature was 27.4 ˚C. The faster growth in warmer temperatures could lead to a shorter pelagic duration for lionfish larvae in the nGoM, as they may be able to exit the vulnerable larval stage complete larval development faster [68]. Future studies should investigate specific links between larval lionfish growth rates and ambient environmental conditions.

## Conclusion

This study represents the first effort to characterize the larval ecology of invasive lionfish in the western Atlantic. We demonstrate that while geographic location and sampling year were the most important factors determining the distribution of lionfish larvae, other environmental and temporal factors are also related to larval lionfish presence. Larvae were generally found in comparatively warm water in the week following the full moon, and variable detection probabilities over the course of the day suggest that lionfish larvae are capable of altering their behavior between the day and night. The factors examined in this study explained only a small amount of the observed variation in lionfish presence, suggesting that lionfish larvae are not constrained by narrow environmental tolerances in the invaded region. These findings highlight a question that has not yet been addressed in the context of the lionfish invasion. Given the highly disparate conditions experienced by the larval and adult stages of many marine organisms, how does the ecology of the pelagic larval stage affect the invasive potential of a species? Several avenues of future research may shed light on this topic. First, while this study found that warmer waters were more frequently associated with larval lionfish presence, it is unknown whether warmer waters lead to increased larval survival and juvenile recruitment rates in lionfish, or if larval success is largely a function of other factors. The physiological and developmental impacts of warmer water temperatures on lionfish larvae should receive particular focus, as warming ocean temperatures may widen the potential dispersal window and shorten larval durations in this species [82]. We also suggest comparing the growth rates, mortality and pelagic habitat distribution of lionfish larvae with those of native fish larvae, especially species with a similar trophic role and reproductive schedule to lionfish. Finally, additional research on the larval ecology of *Pterois* spp. in their native range would provide a baseline against which larval lionfish habitat characteristics in the invaded range could be compared. These studies will reveal if invasive lionfish larvae possess ecological advantages or disadvantages relative to native fish larvae, which in turn would provide insight into whether the bipartite life cycle of the lionfish was a promotive, prohibitive or neutral factor in the spread of the invasion.

## Supporting information

S1 Table Summary of ichthyoplankton cruise dates, vessels, and sampling bounds. (DOCX)

S2 Table Ichthyoplankton collection stations compiled for this study. Includes the values of the environmental variables used to construct GAMs. Only stations with entries for all environmental variables were included in the GAM construction dataset.
(XLSX)

S3 Table Gear deployment records from all ichthyoplankton collection cruises compiled for this study. (XLSX)

S4 Table Larval lionfish collection records compiled for this study. Cruise codes correspond to the cruises in S1 Table. N/A indicates missing or inapplicable data.
(XLSX)
