# Supplementary material for: Early life ecology of the invasive lionfish (Pterois spp.) in the western Atlantic
Source: PLoS One. 2020 Dec 10;15(12):e0243138. doi: 10.1371/journal.pone.0243138 (PMC7728204; doi:10.1371/journal.pone.0243138)
Supplement: S1 Table — (DOCX) [file pone.0243138.s001.docx]

| **Cruise Name** | **Year** | **Start Date** | **End Date** |  | **Vessel** | **Longitude Minimum** | **Longitude Maximum** | **Latitude Minimum** | **Latitude Maximum** |
| --- | --- | --- | --- | --- | --- | --- | --- | --- | --- |
| WS0921 | 2009 | 23-Nov | 24-Nov |  | *R/V F.G. Walton Smith* | -79.931 | -79.202 | 27.000 | 27.003 |
| GU1001 | 2010 | 7-Apr | 21-May |  | *R/V Gordon Gunter* | -96.253 | -83.500 | 19.488 | 29.983 |
| WS1009 |  | 19-May | 20-May |  | *R/V F.G. Walton Smith* | -79.931 | -79.285 | 27.002 | 27.007 |
| NF1013 |  | 1-Jul | 18-Jul |  | *R/V Nancy Foster* | -88.451 | -81.991 | 23.219 | 29.999 |
| WS1016 |  | 23-Aug | 24-Aug |  | *R/V F.G. Walton Smith* | -79.927 | -79.198 | 27.000 | 27.008 |
| GU1004 |  | 25-Aug | 28-Sep |  | *R/V Gordon Gunter* | -94.516 | -81.573 | 24.499 | 30.327 |
| GU1101 | 2011 | 31-Mar | 27-May |  | *R/V Gordon Gunter* | -96.003 | -73.512 | 16.175 | 29.981 |
| WS1109 |  | 14-Jun | 15-Jun |  | *R/V F.G. Walton Smith* | -79.930 | -79.201 | 26.994 | 27.001 |
| WS1114 |  | 21-Sep | 22-Sep |  | *R/V F.G. Walton Smith* | -79.931 | -79.200 | 27.000 | 27.005 |
| GU1201 | 2012 | 2-Apr | 28-May |  | *R/V Gordon Gunter* | -96.008 | -79.270 | 15.943 | 29.981 |
| WS1206 |  | 29-May | 30-May |  | *R/V F.G. Walton Smith* | -79.928 | -79.200 | 26.987 | 27.002 |
| WS1210 |  | 13-Jul | 14-Jul |  | *R/V F.G. Walton Smith* | -79.932 | -79.200 | 26.997 | 27.006 |
| GU1204 |  | 10-Oct | 18-Nov |  | *R/V Gordon Gunter* | -96.497 | -84.011 | 24.993 | 29.789 |
| ORII303 | 2013 | 30-Apr | 29-May |  | *R/V Oregon II* | -96.020 | -83.492 | 23.988 | 29.992 |
| NF1304 |  | 2-May | 22-May |  | *R/V Nancy Foster* | -81.508 | -73.987 | 24.231 | 30.007 |
| PS1305 |  | 22-Aug | 24-Sep |  | *R/V Pisces* | -97.199 | -81.689 | 25.000 | 30.326 |
| NF1502 | 2015 | 11-Apr | 31-May |  | *R/V Nancy Foster* | -87.379 | -64.371 | 17.525 | 23.628 |
| ORII317 | 2016 | 30-Apr | 30-May |  | *R/V Oregon II* | -96.011 | -83.488 | 23.999 | 29.995 |
| NF1602 |  | 10-May | 15-Jun |  | *R/V Nancy Foster* | -87.361 | -63.391 | 17.727 | 24.004 |
